# Revisiting Mitochondrial pH with an Improved Algorithm for Calibration of the Ratiometric 5(6)-carboxy-SNARF-1 Probe Reveals Anticooperative Reaction with H^+^ Ions and Warrants Further Studies of Organellar pH

**DOI:** 10.1371/journal.pone.0161353

**Published:** 2016-08-24

**Authors:** Tomasz Michał Żurawik, Adam Pomorski, Agnieszka Belczyk-Ciesielska, Grażyna Goch, Katarzyna Niedźwiedzka, Róża Kucharczyk, Artur Krężel, Wojciech Bal

**Affiliations:** 1 Institute of Biochemistry and Biophysics, Polish Academy of Sciences, Pawińskiego 5a, 02-106, Warsaw, Poland; 2 Department of Chemical Biology, Faculty of Biotechnology, University of Wrocław, F. Joliot-Curie 14A, 50-383, Wrocław, Poland; Instituto Nacional de Cardiologia Ignacio Chavez, MEXICO

## Abstract

Fluorescence measurements of pH and other analytes in the cell rely on accurate calibrations, but these have routinely used algorithms that inadequately describe the properties of indicators. Here, we have established a more accurate method for calibrating and analyzing data obtained using the ratiometric probe 5(6)-carboxy-SNARF-1. We tested the implications of novel approach to measurements of pH in yeast mitochondria, a compartment containing a small number of free H^+^ ions. Our findings demonstrate that 5(6)-carboxy-SNARF-1 interacts with H^+^ ions inside the mitochondria in an anticooperative manner (Hill coefficient *n* of 0.5) and the apparent pH inside the mitochondria is ~0.5 unit lower than had been generally assumed. This result, at odds with the current consensus on the mechanism of energy generation in the mitochondria, is in better agreement with theoretical considerations and warrants further studies of organellar pH.

## Introduction

In a recent conceptual paper, we analyzed the limits of the classical definition of pH in very small volumes. We concluded that due to a poor ability of water molecules to autodissociate, expressed by the ionic product of water, very few free H^+^ ions can ever be present in femtoliter and lower volumes, which characterize cellular compartments. In particular, according to our calculations, the number of such free H^+^ ions in the mitochondrial matrix and the intermembrane space is on average 3.4 and 6.7 H^+^ ions, respectively [1]. These very low numbers are apparently at odds with the current understanding of the molecular machinery of mitochondrial energy generation, which is based on the H^+^ ion gradient across the mitochondrial inner membrane [2]. In order to reconcile the results of our analysis with this otherwise very well established view, we first noted the fact that molecular probes used for such measurements actually report their own protonation state rather than the concentration of free protons in solution. They can be protonated/deprotonated by interactions with other H^+^ ion exchanging molecules, which we can call “proton chaperones”, and not necessarily by freely diffusing H^+^ ions. We suggested that due to their abundance and p*K*_a_ values close to 7, such a role can be played by inorganic phosphates (H_2_PO_4_^-^/HPO_4_^2-^), nucleotides and phospholipids composing the biological membranes. This concept was indirectly supported by an independent study, which demonstrated that phosphates, rather than amino acid side chains on the surface of proteins, constitute the main pH buffer in eukaryotic cells [3].

This view has several important consequences. One of them is that individual molecular pH sensors may behave in the biological compartment in a different way than in a bulk solution. In order to corroborate this view, we performed the sensor calibration in the bulk solution and in the matrix of living mitochondria, followed by pH determination in the matrix using 5(6)-carboxy-SNARF-1 (mixture of 5- and 6- isomers, in brief carboxy-SNARF-1), a commercially available and commonly used ratiomeric fluorescent pH indicator [4].

**Fig 1:**
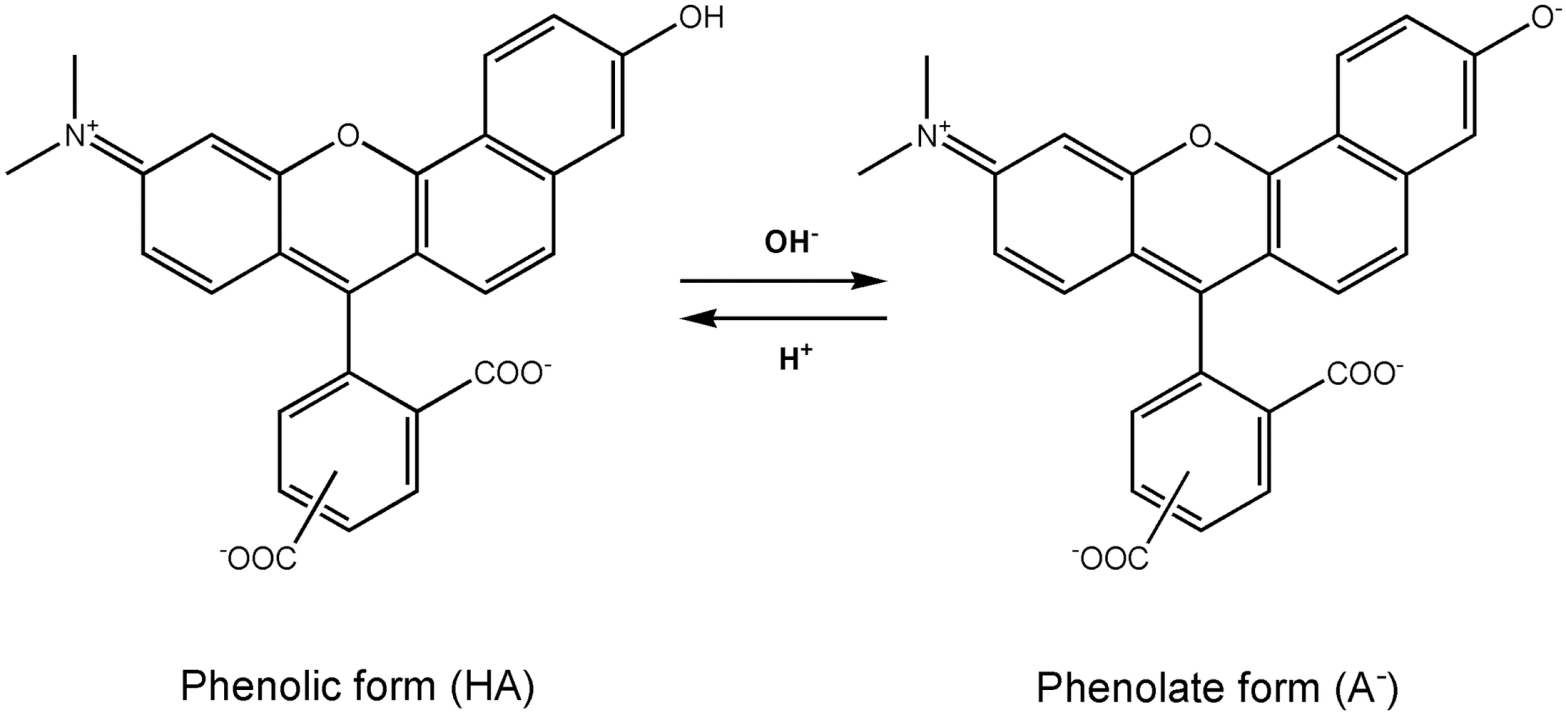
Structures of the phenolic (HA) and phenolate (A^-^) forms of 5(6)-carboxy SNARF-1 responsible for ratiometric properties of the probe. For clarity, only the deprotonation of the phenolic group is shown.

Carboxy-SNARF-1 is one of the two most widely used pH-probes for the detection of pH in intact cells and cellular compartments, beside BCECF (2',7'-*bis*-(2-carboxyethyl)-5(6)-carboxyfluorescein). Among others, it was used for the determination of absolute pH values of cytosol [5–7], mitochondria [8] and cell nucleus [5]. It was applied to living cells, flow cytometry [9], microplate readers [10], confocal imaging [8] and microspectrofluorimetry [11]. The pH-reporting property of carboxy-SNARF-1 is based on its unique chemical structure and physicochemical behavior. The fluorescent platform of carboxy-SNARF-1 is asymmetric, as it is built in half by naphthofluorescein and in half by the tetramethylrhodamine probe. Because both of these fluorescent probes demonstrate different emission properties, carboxy-SNARF-1 exhibits two independent emission bands. Fig 1 presents the structure of carboxy-SNARF-1 where the protonated state (phenolic state HA) has a fluorescence maximum wavelength at ca. 580 nm, while the deprotonated state (phenolate state A^-^) has a fluorescence maximum wavelength at ca. 640 nm, which corresponds to the naphthofluorescein behavior. These properties enable measurement of the ratio of protonated and deprotonated carboxy-SNARF-1 species without the necessity of knowing its actual concentration in the studied biological compartment (ratiometric principle). The chemical stability, resistance to photobleaching and the range of emission that is free of interference from other components of the biological milieu makes carboxy-SNARF-1 a probe of choice for experiments in intact mitochondria.

## Experimental Procedures

### Reagents

5-(6)-carboxy-SNARF-1 and its membrane-permeable derivative 5-(6)-carboxy-SNARF-1 acetoxymethyl ester (carboxy-SNARF-1-AM) were obtained from ThermoFisher. Carbonyl cyanide 3-chlorophenylhydrazone (CCCP) and other reagents were obtained from Sigma-Aldrich.

### Isolation of mitochondria

Native mitochondria were prepared with enzymatic method as described earlier [12] from the MR6 (*Mata ade2-1 his3-11*,*15 trp1-1 leu2-3*,*112 ura3-1 arg8*::*HIS3 ρ+ WT*) yeast cells strain grown to the mid exponential phase (3–4 × 10^7^ cells ml^–1^) in YPGAla medium [13]. The final mitochondrial pellet was resuspended in a ‘respiration buffer’ (0.65 M mannitol, 0.36 mM EGTA, 5 mM Tris-phosphate, 10 mM Tris-maleate, pH 6.8). Protein concentrations were determined with the Lowry procedure in the presence of 5% SDS [14]. The mitochondria were tested for viability by measuring oxygen consumption and mitochondrial inner membrane potential stability as described previously [15].

### Calibration of carboxy-SNARF-1 in vitro

The calibration of carboxy-SNARF-1 in the bulk solution was done in two modes. In the first mode, the 20 μM solution of carboxy-SNARF-1 in the ‘respiration buffer’ was manually titrated in the pH range of 6.35‒10.41 by adding minute portions of concentrated HCl or NaOH and determining the resulting pH using a pH-meter equipped with a glass electrode. In the second mode, analogous experiment was performed in the presence of mitochondria suspension (OD_600_ = 0.3), in order to account for light scattering by the mitochondria. The fluorescence spectra were recorded on a Cary Eclipse spectrofluorometer in the 520–720 nm wavelength range, at a scan rate of 60 nm/min, following excitation at 488 nm, using 5 nm excitation slit and 10 nm emission slit of.

### Calibration of carboxy-SNARF-1 in the mitochondrial matrix

Prepared mitochondria were incubated with 20 μM carboxy-SNARF-1 acetoxymethyl ester (SNARF-1 AM) for 30 min in the ‘respiration buffer’ at 30°C. The usage of the acetoxymethyl ester (membrane penetrable) form of carboxy-SNARF-1 undergoing hydrolysis to the acidic form (membrane impenetrable) by intramitochondrial esterases, assured that the measurement was performed in the matrix of mitochondria [16].

The mitochondria staining and kinetic diffusion experiments were performed with SNARF-1 AM in order to assure that all fluorescence detected would originate from the probe immobilized inside the matrix of mitochondria. The staining protocol was the same as above. The mitochondria were washed twice and resuspended in the ‘respiration buffer’ after each staining. There were no significant differences between the fluorescence emission spectra obtained for the mitochondria stained with carboxy-SNARF-1 and for control samples of mitochondria which were not stained (background emissions in both cases).

The kinetic diffusion experiment was carried out directly after SNARF-1 AM staining and after 10, 20 and 30 minute incubations at room temperature. Mitochondria were centrifuged down and the fluorescence emission spectra of the supernatant buffer were measured. The time used for sample preparation before the measurement was about 15 minutes in all experiments (including calibration experiments described below) and in the course of the kinetic experiment no significant diffusion of carboxy-SNARF-1 (hydrolyzed form) from the matrix of the mitochondria was observed during that time.

These control experiments provided us with evidence that the SNARF-1 AM staining was stable and selective for the matrix of mitochondria.

The calibration of carboxy-SNARF-1 in the mitochondria was enabled by application of 4 μM CCCP which equalizes the pH value between the buffer and the matrix of mitochondria [17, 18]. The fluorescence spectra were recorded in the pH range of 2.84–10.60, on a Cary Eclipse spectrofluorometer, using the same settings as above. The experiments were performed in three repetitions, each time using mitochondria from different preparations.

### Determination of pH in mitochondria

The mitochondria staining protocol and the fluorescence measurement parameters were analogous to those described in the previous paragraph. The fluorescence spectra were obtained in the buffer pH range (4.82–10.96) which, according to the literature, does not affect the pH value in the mitochondrial matrix. At low pH values (2.8–4.8) the inner membrane of the mitochondria became proton-permeable, while still non-permeable to the probe. The experiments were performed in three repetitions, using mitochondria from different preparations.

### Data analysis

All experimental and further transformed data were fitted to appropriate equations listed and discussed in the Results section below, using the non-linear least square fitting module of the OriginPro 8.1 software. The analysis of data using Eqs 9, 10, 12, 14 and 17 required fixing of the limiting values of fluorescence intensity at the appropriate wavelengths for the phenolic and phenolate species, which corresponds to the limiting values of Eq 7. It should be noted that even though the equations listed below are not in the logarithmic form, they can process the logarithmic data (pH) thanks to the appropriate annotation in the fitting function (e.g. in the OriginPro 8.1 software).

## Results

### Calculation of carboxy-SNARF-1 *K*_a_ in bulk solution

**Fig 2:**
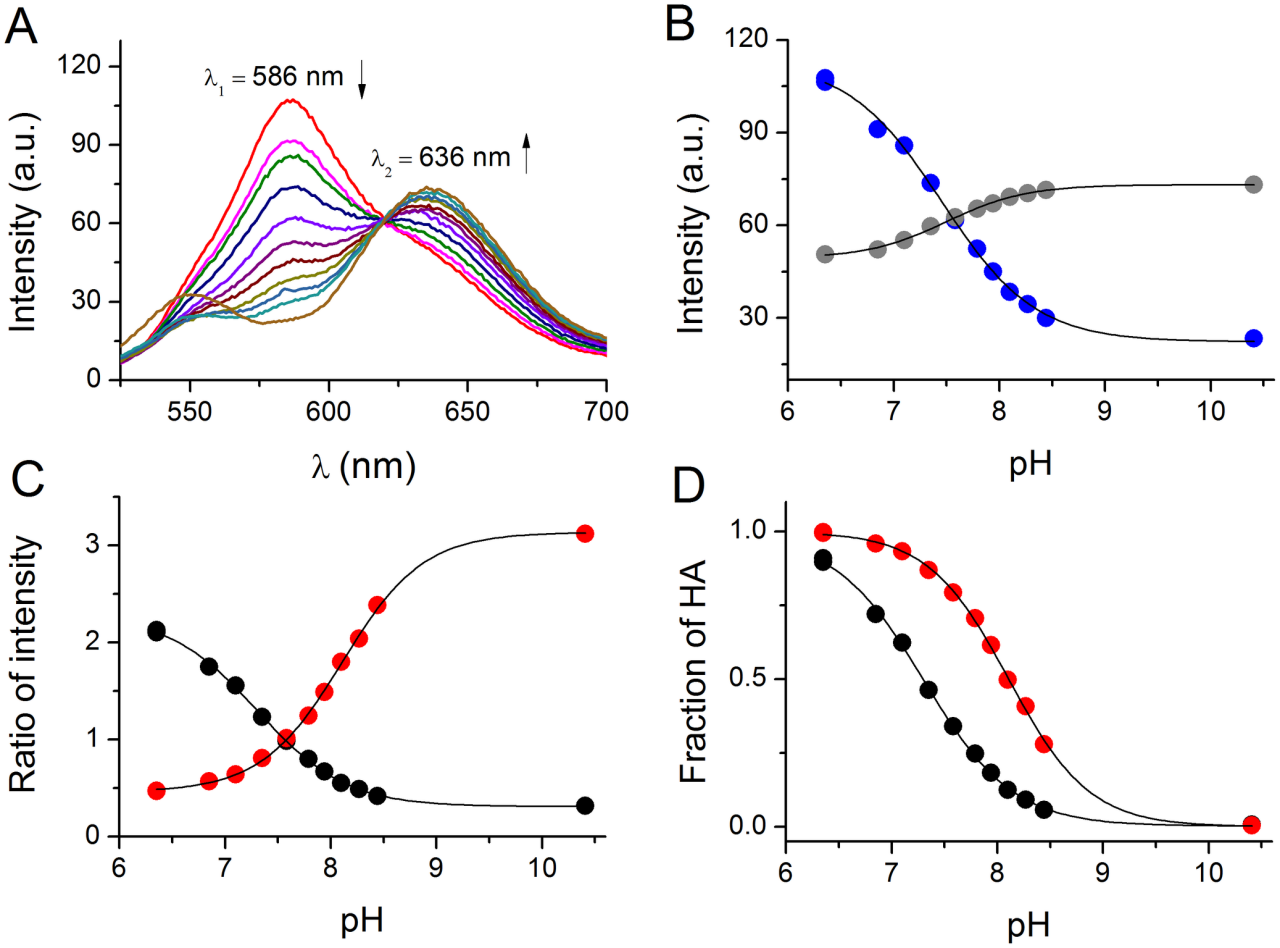
**Calibration of carboxy-SNARF-1 in pH-controlled buffers**; A. Emission spectra recorded in the pH range of 6.35–10.41; B. Fluorescence intensities at 586 nm (λ_1_—blue) and 636 nm (λ_2_—grey) at different pH values, fitted to Eq 6; C. Changes of the fluorescence ratios R_12_ (red) and R_21_ (black) at different pH values, fitted to Eq 5; D. Comparison of the molar fractions of protonated phenolic HA species derived either from the R_12_ or the R_21_ molar ratios from Fig 2C.

**Fig 3:**
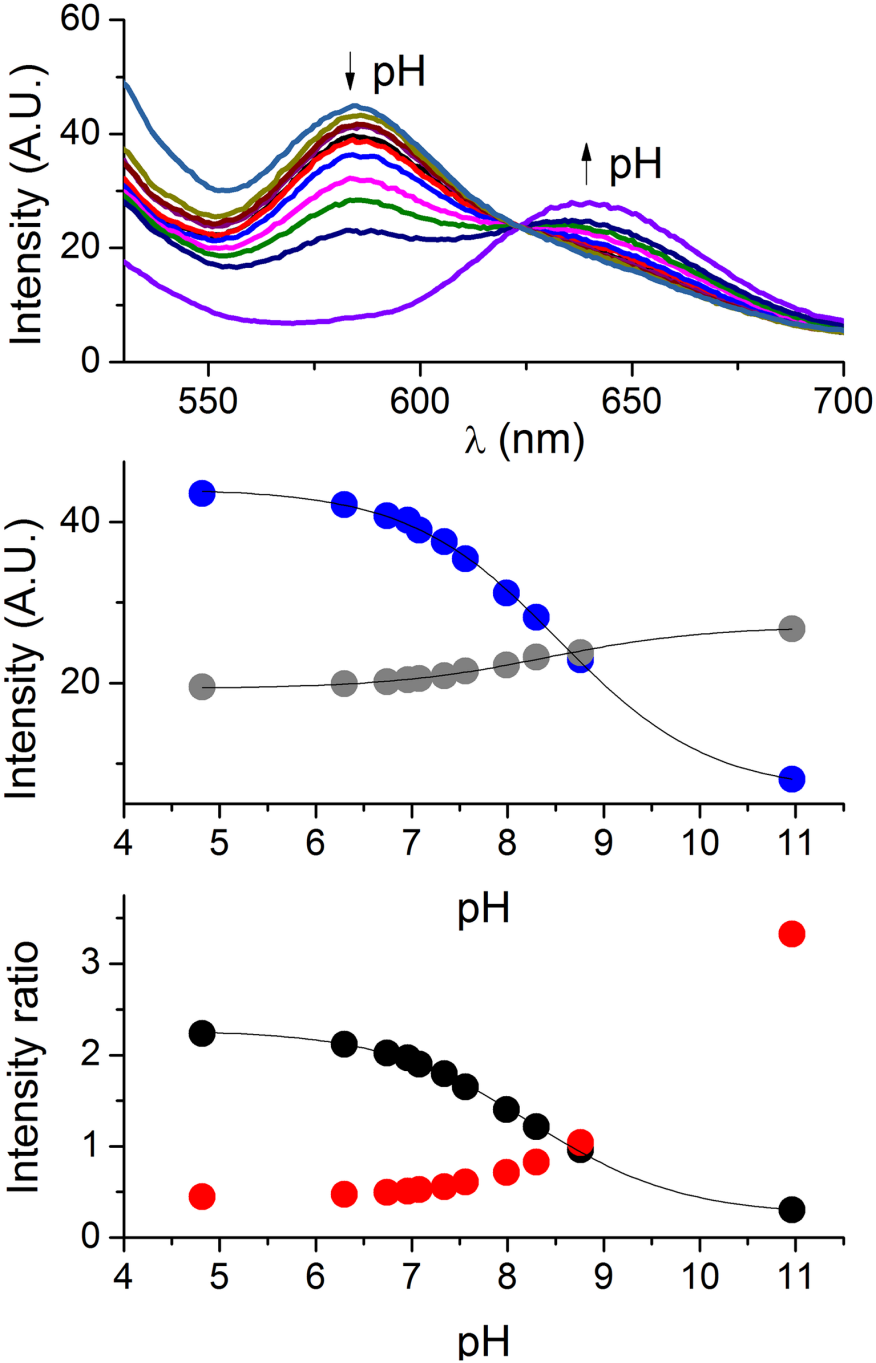
**Calibration of SNARF-1 in the mitochondrial matrix in pH-controlled buffers**; A. Emission spectra recorded in the pH range 4.82‒10.96; B. Fluorescence intensities at 586 nm (λ_1_—blue) and 636 nm (λ_2_—grey) at different pH values fitted to Eq 7; C. Changes of the fluorescence ratio R_12_ (red) and R_21_ (black) at different pH values and the fit to Eq 10 for R_21_. The R_12_ ratio data could not be fitted to the equation since there is no plateau in the high pH range and the endpoint could not be precisely established.

Fig 2A presents the emission spectra of carboxy-SNARF-1 recorded at different pH values of the ‘respiration buffer’ (bulk solution conditions). The spectra exhibited two bands, one at 586 nm decreasing as a function of increasing pH and another one at 636 nm, exhibiting an opposite behavior. The isosbestic point was present at 620 nm for the whole pH range of titration. The maximum wavelengths agree well with those described in the probe’s manual [19]. Importantly, these values remained unchanged when carboxy-SNARF-1 was used for the measurements in mitochondria (Fig 3A).

Several steps of the procedure of conversion of fluorescence intensity changes at particular wavelengths into pH values have to be considered precisely. Recently, we demonstrated that the way in which ratiometric data are processed may significantly affect the outcome of calculations [20]. Furthermore, as we show below, the acid dissociation constant of carboxy-SNARF-1, *K*_a_ must be determined in conditions that are as similar as possible to those used for the biological pH determination. Moreover, the cooperativity coefficient (Hill parameter *n*) must also be taken under consideration.

The dissociation of phenolic carboxy-SNARF-1 to its phenolate form occurs according to Eq 1 and can be described by the acid dissociation constant *K*_a_ in Eq 2.

$$HA\;\left(phenolic\right)\;\;⇄A^{-}\;\left(phenolate\right)\;+\;H^{+}$$ (1)

$$K_{\text{a}}=\frac{\left[{\text{A}}^{-}\right]\left[{\text{H}}^{+}\right]}{\left[\text{HA}\right]}$$ (2)

For a ratiometric probe such as carboxy-SNARF-1, one can define R as the ratio of intensity of fluorescence at λ_1_ (586 nm) to λ_2_ (636 nm) at a particular pH value. Then molar concentrations of [A^-^] and [HA] are R_a_ − R and R − R_b_, respectively, where R_a_ and R_b_ are the limiting fluorescence values at acidic and basic conditions. Then the probe *K*_a_ and [H^+^] can be described by Eqs 3 and 4.

$$K_{\text{a}}=\frac{\left({\text{R}}_{\text{a}}-\text{R}\right)\;\left[{\text{H}}^{+}\right]}{\left(\text{R}-{\text{R}}_{\text{b}}\right)}$$ (3)

$$\left[{\text{H}}^{+}\right]=\;K_{\text{a}}\frac{\left(\text{R}-{\text{R}}_{\text{b}}\right)}{\left({\text{R}}_{\text{a}}-\text{R}\right)}$$ (4)

Transforming Eq 4 with respect to R allows one to calculate the *K*_a_ value from a pH-dependent calibration experiment where R is the ordinate (Eq 5).

$$\text{R}={\text{R}}_{\text{a}}\frac{\left[{\text{H}}^{+}\right]}{K_{\text{a}}+\left[{\text{H}}^{+}\right]}+{\text{R}}_{\text{b}}\frac{K_{\text{a}}}{K_{\text{a}}+\left[{\text{H}}^{+}\right]}$$ (5)

The application of Eq 5 to the experimental data presented in Fig 2A yielded *K*_a_ values differing significantly for R defined either as I_λ1_/I_λ2_ (R_12_) or I_λ2_/I_λ1_ (R_21_), where I is the intensity of fluorescence at a given wavelength. The traditional way of treating such data makes use of a classical one binding site equation (Eq 6) applied to data obtained at a single wavelength, which allows one to calculate *K*_a_ values independently from intensity changes of both λ_1_ and λ_2_. In Eq 6 I_a_ and I_b_ are limiting values of fluorescence intensities in acidic and basic conditions (Fig 2B). However, if the deprotonation reaction can be affected by any additional interactions introduced by the medium where the measurement takes place or depends on other chemical process, then the cooperativity index *n* (also known as Hill coefficient) different from 1 should be included during data analysis. Eq 6 should therefore be expanded by the introduction of index *n* (Eq 7).

$$\text{I}={\text{I}}_{\text{a}}\frac{\left[{\text{H}}^{+}\right]}{K_{\text{a}}+\left[{\text{H}}^{+}\right]}+{\text{I}}_{\text{b}}\frac{K_{\text{a}}}{K_{\text{a}}+\left[{\text{H}}^{+}\right]}$$ (6)

$$\text{I}={\text{I}}_{\text{a}}\frac{{\left[{\text{H}}^{+}\right]}^{n}}{K_{\text{a}}^{n}+{\left[{\text{H}}^{+}\right]}^{n}}+{\text{I}}_{\text{b}}\frac{K_{\text{a}}^{n}}{K_{\text{a}}^{n}+{\left[{\text{H}}^{+}\right]}^{n}}$$ (7)

$$\text{R}={\text{R}}_{\text{a}}\frac{{\left[{\text{H}}^{+}\right]}^{n}}{K_{\text{a}}^{n}+{\left[{\text{H}}^{+}\right]}^{n}}+{\text{R}}_{\text{b}}\frac{K_{\text{a}}^{n}}{K_{\text{a}}^{n}+{\left[{\text{H}}^{+}\right]}^{n}}$$ (8)

Table 1 includes the *K*_a_ (in the more convenient logarithmic p*K*_a_ form) and *n* values for fitting procedures performed for the calibration of carboxy-SNARF-1 in bulk solution conditions according to various equations. The p*K*_a_ values obtained by fitting the signal intensities to Eqs 6 and 7 are almost the same, as the cooperativity index *n* is close to 1. This, however, does not explain the hugely different values obtained from ratiometric calculations (Eq 5; Fig 2C and 2D). There, even the introduction of Hill coefficient *n* to the equation (Eq 8) did not help to obtain comparable p*K*_a_ values. This discrepancy was at the foundation of our recent work where we demonstrate that the linear functions of the changes of signal intensity at two different wavelengths of the same ratiometric system have different slopes, and therefore the ratio value is not linear [20]. This non-linearity of the ratio function introduces a serious error in the data analysis. In order to cope with non-linear effects, we developed equations that use signal intensities at particular wavelengths at their limiting values. These equations also utilize the Hill coefficient *n* (Eqs 9 and 10 [18]).

**Table 1:** The comparison of p*K*_a_ values and Hill coefficients *n* obtained from the carboxy-SNARF-1 calibration *in vitro* by fitting intensities at different wavelengths and ratios to various equations discussed. Eqs 8 and 9 were derived in our previous study [20]; *n/a*–not applicable (*n* is not present in the equation). Eqs 12 and 14 were proposed by the probe manufacturer [19].

Fitting method	Equation	p*K*_a_	*n*
λ_1_/λ_2_—R_12_	Eq 5 (modified Henderson-Hasselbalch)	7.29 ± 0.02	*n/a*
λ_2_/λ_1_—R_21_	Eq 5 (modified Henderson-Hasselbalch)	8.11 ± 0.04	*n/a*
λ_1_/λ_2_—R_12_	Eq 8 (modified Henderson-Hasselbalch with *n* coefficient)	7.29 ± 0.02	1.01 ± 0.04
λ_2_/λ_1_—R_21_	Eq 8 (modified Henderson-Hasselbalch with *n* coefficient)	8.11 ± 0.01	1.14 ± 0.04
λ_1_	Eq 6 (one-site binding)	7.46 ± 0.03	*n/a*
λ_2_	Eq 6 (one-site binding)	7.48 ± 0.04	*n/a*
λ_1_	Eq 7 (one-site binding with *n* coefficient)	7.45 ± 0.03	0.98 ± 0.06
λ_2_	Eq 7 (one-site binding with *n* coefficient)	7.50 ± 0.06	1.15 ± 0.08
λ_1_/λ_2_—R_12_	Eq 9 (our equation)	7.44 ± 0.02	0.98 ± 0.03
λ_2_/λ_1_—R_21_	Eq 10 (our equation)	7.48 ± 0.02	1.06 ± 0.05
R_12_	Eq 12 (carboxy-SNARF-1 manual)	7.46 ± 0.02	*n/a*
R_21_	Eq 14 (carboxy-SNARF-1 manual)	7.40 ± 0.02	*n/a*

$${\text{R}}_{12}=\frac{{\text{I}}_{1\text{a}}{\left[{\text{H}}^{+}\right]}^{n}+{\text{I}}_{1\text{b }}K_{\text{a}}^{n}}{{\text{I}}_{2\text{a}}{\left[{\text{H}}^{+}\right]}^{n}+{\text{I}}_{2\text{b}}K_{\text{a}}^{n}}$$ (9)

$${\text{R}}_{21}=\frac{{\text{I}}_{2\text{a}}{\left[{\text{H}}^{+}\right]}^{n}+{\text{I}}_{2\text{b }}K_{\text{a}}^{n}}{{\text{I}}_{1\text{a}}{\left[{\text{H}}^{+}\right]}^{n}+{\text{I}}_{1\text{b}}K_{\text{a}}^{n}}$$ (10)

In these equations I_1b_ and I_2b_ are the intensities of fluorescence at λ_1_ and λ_2_, respectively, in basic conditions and I_1a_ and I_2a_ are the intensities of fluorescence at λ_1_ and λ_2_, respectively, in acidic conditions. The application of these equations to ratiometric data for carboxy-SNARF-1 resulted in a p*K*_a_ value comparable to those calculated from changes of fluorescence signal at a single wavelength.

In order to compare different computational approaches to the issue of carboxy-SNARF-1 calibration we also utilized the equation presented in the Molecular Probes manual for this probe (Eq 11) [19]. Its transformation with respect to R_12_ yielded Eq 12, suitable for fitting the data from ratiometric measurements but provided only the formula for the application of R_12_, nonetheless, it is easy to infer that a similar equation utilizing R_21_ can be used. It only requires the F_1b_/F_1a_ factor to be included in the formula (Eq 13). Its transformation for R_21_ as the ordinate is given in Eq 14.

$$\left[{\text{H}}^{+}\right]=\;K_{\text{a}}\frac{\left({\text{R}}_{12}-{\text{R}}_{\text{b}}\right)}{\left({\text{R}}_{\text{a}}-{\text{R}}_{12}\right)}\;\frac{{\text{F}}_{2\text{b}}}{{\text{F}}_{2\text{a}}}$$ (11)

$${\text{R}}_{12}=\frac{\left[{\text{H}}^{+}\right]{\text{R}}_{\text{a}}{\text{F}}_{2\text{a}}+K_{a}{\text{R}}_{\text{b}}{\text{F}}_{2\text{b}}}{K_{\text{a}}{\text{F}}_{2\text{b}}+\left[{\text{H}}^{+}\right]{\text{F}}_{2\text{a}}}$$ (12)

$$\left[{\text{H}}^{+}\right]=\;K_{\text{a}}\frac{\left({\text{R}}_{21}-{\text{R}}_{\text{b}}\right)}{\left({\text{R}}_{\text{a}}-{\text{R}}_{21}\right)}\;\frac{{\text{F}}_{1\text{b}}}{{\text{F}}_{1\text{a}}}$$ (13)

$${\text{R}}_{21}=\frac{\left[{\text{H}}^{+}\right]{\text{R}}_{\text{a}}{\text{F}}_{1\text{a}}+K_{\text{a}}{\text{R}}_{\text{b}}{\text{F}}_{1\text{b}}}{K_{\text{a}}{\text{F}}_{1\text{b}}+\left[{\text{H}}^{+}\right]{\text{F}}_{1\text{a}}}$$ (14)

### Determination of K_a_ value of carboxy-SNARF-1 in the mitochondrial matrix

Next, we performed experiments on isolated, respiratory active yeast mitochondria. Previously it was stressed that the carboxy-SNARF-1 fluorescence signal must be thoroughly calibrated for a correct pH value calculation [21]. We followed this advice and contrary to the majority of publications we broadened the pH range used for calibration (from 4.82 to 10.96). This approach helped us to establish which computational approach among those presented above was appropriate for the pH determination in the small volume of mitochondrial matrix.

**Fig 4:**
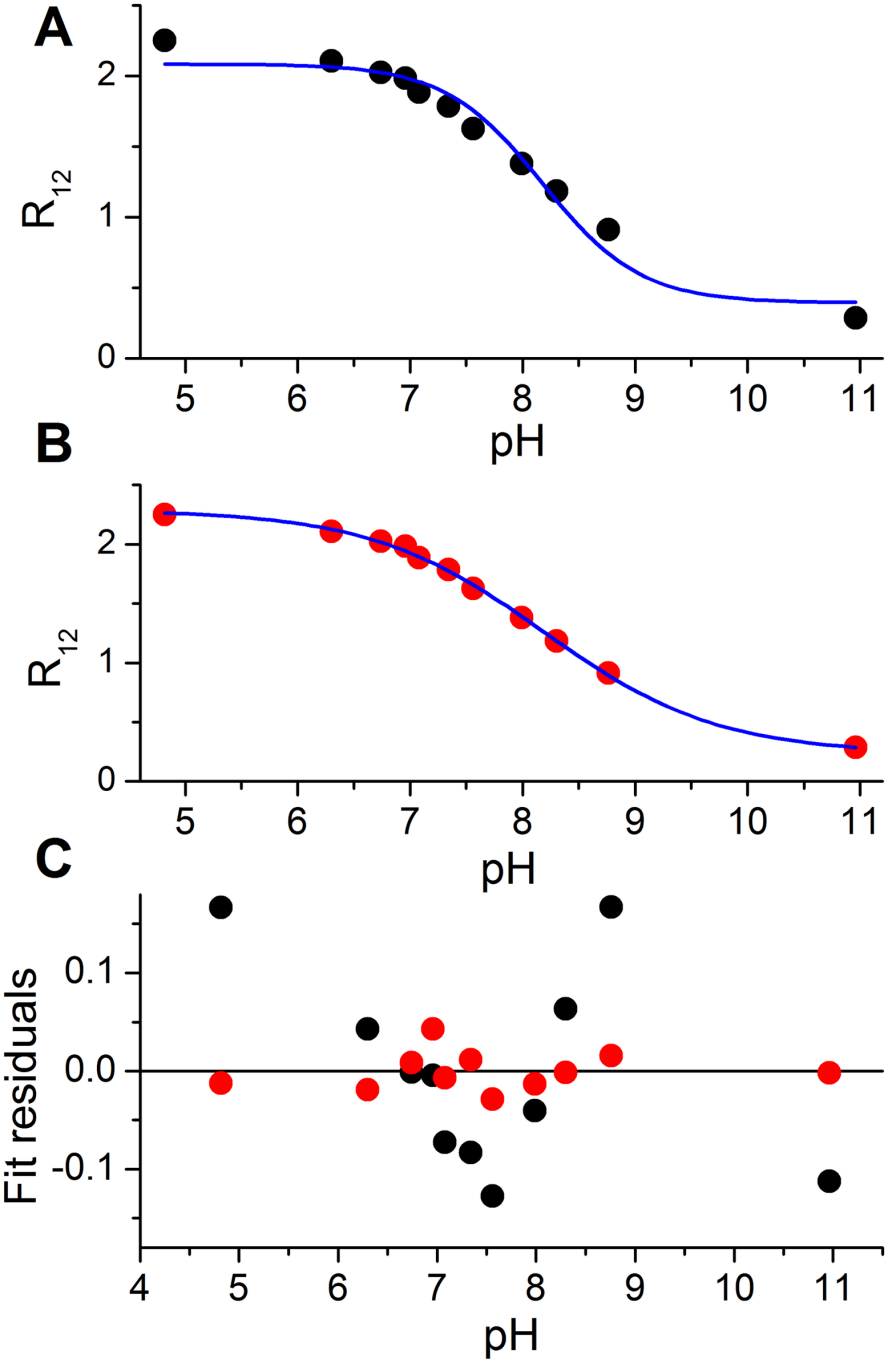
Comparison of the R_12_ fitting data using A. the equation from the carboxy-SNARF-1 manufacturer manual (Eq 12) which does not account for cooperativity; B. Eq 9 with explicit cooperativity coefficient *n*; C. The comparison of residuals of fits A (black dots) and B (red dots).

The exemplary fluorescence spectra obtained for carboxy-SNARF-1 inside the suspension of isolated mitochondria are presented in 3A. All three datasets are presented in S1 Fig. We analyzed these calibration data in a fashion similar to that used for the buffered bulk solutions. The first approach was based on the analysis of the fluorescence intensity changes at single wavelengths corresponding to fluorescence maxima of the phenolate and phenolic species (Fig 3B). Then we performed calculations based on the fluorescence ratio (Fig 3C). In the course of data analysis, it became apparent that the fitting using equations which did not include the cooperativity coefficient *n* produced standard errors much higher than the equations containing *n*. Also, as can be seen in Fig 4, the data points did not converge on the fitted line in the absence of *n* in the equation. Therefore the data in Fig 3B and 3C were fitted using equations accounting for the cooperativity coefficient *n*.

Regardless of the computational method applied, the p*K*_a_ value calculated for carboxy-SNARF-1 in the mitochondrial matrix was significantly higher than the value obtained in the bulk solution. The use of our recently developed equation (Eq 9) resulted in the p*K*_a_ value of 7.44 with *n* = 0.98 for the bulk conditions, while the same equation yielded the average p*K*_a_ value of 8.51 with *n* = 0.54 for the mitochondria. The same effect was seen when, traditionally, the ratio values were used directly (Eq 5). Furthermore, the p*K*_a_ value calculated from Eq 5 was different from the value calculated from intensities at particular wavelengths (Eq 6). It also should be noted here that the equation proposed in the manufacturer’s manual (Eq 12) yielded an inaccurate value of p*K*_a_, because there is no *n* coefficient in the equation. Full data analysis using different equations is presented in Table 2.

**Table 2:** The comparison of p*K*_a_ values and *n* coefficients obtained from the carboxy-SNARF-1 calibration in the mitochondrial matrix by fitting of spectral intensities at various wavelengths and intensity ratios to various equations described in the text. Data for three independent determinations (data sets 1, 2 and 3) are given explicitly. Values marked with italics have significantly larger errors compared to other calculation methods using explicit *n* value fitting.

Fitting method	Equation	p*K*_a_	*n* coefficient
λ_1_/ λ_2_—R_12_	Eq 8	8.19 ± 0.03 8.29 ± 0.04 8.24 ± 0.03	0.57 ± 0.02 0.57 ± 0.03 0.52 ± 0.02
λ_2_/ λ_1_—R_21_	Eq 8	*n/d*	*n/d*
λ_1_/ λ_2_—R_12_	Eq 5	8.18 ± 0.11 8.25 ± 0.11 8.23 ± 0.12	*n/a*
λ_2_/ λ_1_—R_21_	Eq 5	*n/d*	*n/a*
λ_1_	Eq 6	8.44 ± 0.09 8.52 ± 0.10 8.46 ± 0.10	*n/a*
λ_2_	Eq 6	8.33 ± 0.11 8.37 ± 0.10. 8.32 ± 0.16	*n/a*
λ_1_	Eq 7	8.49 ± 0.04 8.60 ± 0.04 8.52 ± 0.03	0.57 ± 0.03 0.55 ± 0.03 0.54 ± 0.02
λ_2_	Eq 7	8.37 ± 0.06 8.42 ± 0.06 8.43 ± 0.16	0.55 ± 0.05 0.59 ± 0.05 0.43 ± 0.07
λ_1_/ λ_2_—R_12_	Eq 9	8.47 ± 0.02 8.57 ± 0.02 8.50 ± 0.02	0.56 ± 0.01 0.56 ± 0.01 0.51 ± 0.01
λ_2_/ λ_1_—R_21_	Eq 10	*n/d*	*n/a*
R_1/2_	Eq 12	8.33 ± 0.11 8.41 ± 0.11 8.36 ± 0.12	*n/a*
R_2/1_	Eq 14	*n/d*	*n/a*

*n/a*–not applicable, *n/d*–not determined due to lack of titration end-point.

### Determination of pH of the mitochondrial matrix based on the in situ calibration

The final step of our investigations was to apply the expertise developed in the above sections to determine the pH in the matrix of isolated yeast mitochondria. For this purpose, we transformed Eqs 9 and 10 so that the proton concentration, [H^+^] could be obtained as the abscissa with either R_21_ or R_12_ providing the ordinate. Thus, Eq 9 was linearized (Eq 15), reordered to have solely [H^+^]^*n*^ on the left side (Eq 16) and finally transformed by applying the *n*-root to its both sides (Eq 17). Eq 9 was converted analogously (Eq 18). In the case of our data, the R_21_ ratio could not be used due to lack of the plateau in the high pH region. Thus, Eq 17 was used to calculate the pH in mitochondria.

$${\left[{\text{H}}^{+}\right]}^{n}\left({\text{R}}_{12}{\text{I}}_{2\text{a}}-{\text{I}}_{1\text{a}}\right)=K_{\text{d}}^{n}\left({\text{I}}_{1\text{b}}-{\text{R}}_{12}{\text{I}}_{2\text{b}}\right)$$ (15)

$${\left[{\text{H}}^{+}\right]}^{n}=K_{\text{a}}^{\text{n}}\frac{{\text{I}}_{1\text{b}}-{\text{R}}_{12}{\text{I}}_{2\text{b}}}{{\text{R}}_{12}{\text{I}}_{2\text{a}}-{\text{I}}_{1\text{a}}}$$ (16)

$$\left[{\text{H}}^{+}\right]=K_{\text{a}}\sqrt[{n}]{\frac{{\text{I}}_{1\text{b}}-{\text{R}}_{12}{\text{I}}_{2\text{b}}}{{\text{R}}_{12}{\text{I}}_{2\text{a}}-{\text{I}}_{1\text{a}}}}$$ (17)

$$\left[{\text{H}}^{+}\right]=K_{\text{a}}\sqrt[{n}]{\frac{{\text{I}}_{2\text{b}}-{\text{R}}_{21}{\text{I}}_{1\text{b}}}{{\text{R}}_{21}{\text{I}}_{1\text{a}}-{\text{I}}_{2\text{a}}}}$$ (18)

It should be noted that, because of the presence of the *n*-th root, the conditions R_12_I_2b_ < I_1b_ and R_12_I_2a_ < I_1a_ must be met to prevent the value under the *n*-th root from being negative.

For the sake of comparison, we calculated the pH in isolated yeast mitochondria using two equations—ours (Eq 17) and that provided by the probe manufacturer (Eq 12). We also tested two different values of carboxy-SNARF-1 p*K*_a_ and *n*. The p*K*_a_ of 8.51 and *n* of 0.54 are the average values calculated using Eq 9, while the average p*K*_a_ of 8.37 was obtained from Eq 12 (*n* is implicitly 1 in this case). The pH value in the matrix of isolated yeast mitochondria was calculated from the intensities ratio using Eq 17. The results are summarized in Table 3.

**Table 3:** The pH values measured using carboxy-SNARF-1 for isolated yeast mitochondria and calculated using various approaches. The average value set in bold typeface represents the correct result obtained from Eq 17 with explicit Hill’s coefficient.

Data set	Eq 17	Eq 17	Eq 12	Eq 12
	p*K*_a_ = 8.51^a^	p*K*_a_ = 8.51^a^	p*K*_a_ = 8.51^a^	p*K*_a_ = 8.37^b^
	*n* = 0.54^a^	*n* = 1		
1	7.12	7.76	7.76	7.62
2	7.04	7.72	7.72	7.58
3	7.61	8.02	8.02	7.88
Average	**7.26 ± 0.31**	7.83 ± 0.16	7.83 ± 0.16	7.69 ± 0.16

^*a*^ the value is the average for p*K*_a_ or *n* from set 1–3 calculated using Eq 9.

^*b*^ the value is the average for p*K*_a_ from set 1–3 calculated using Eq 12.

The data collected in Table 3 indicate that the inclusion of the cooperativity coefficient *n* is a key factor differing the results of two approaches described above.

## Discussion

It is known that ratiometric methods help avoid experimental artifacts in pH determination in biological systems, such as photobleaching, instrument instability, leakage of the probe, changes of cell thickness during their movement, or non-uniform loading of the probe into the cell. Still, the key issues that have to be controlled are the correction of fluorescence background subtraction before the ratio calculation and the correct calibration of the probe − determination of its p*K*_a_ under conditions relevant to a given experiment. The protocol for carboxy-SNARF-1 usage issued by its manufacturer provides its p*K*_a_ value determined in pH-controlled buffers rather vaguely as ~7.5. Nevertheless, it is known that results of calibration performed for this probe inside the cells vary from values determined *in vitro*. For example, in Owen’s work, the determined p*K*_a_ for carboxySNARF-1 was 7.5 in aqueous buffer and 7.7 in mouse spleen [21]. In another example, the p*K*_a_ values of 7.55 in aqueous buffer and 7.75 in Chinese hamster ovarian carcinoma cells were obtained [22]. Our recent findings added another key issue to this list by demonstrating that the standard way of converting the data into ratios yields non-linear effects and biased p*K*_a_ values [20]. The main body of the presented work was therefore devoted to establishing an accurate method of using carboxy-SNARF-1 to measure pH in biological structures. We reconfirmed Owen’s findings [21] that the probe’s p*K*_a_ value determined *in vitro* should not be used for biological targets, such as isolated organelles or cells. Interestingly, we also discovered the need to account for the cooperativity coefficient *n* in the data analysis. Importantly, our results demonstrate that a broad pH range must be used for the proper probe calibration in a given milieu. Without it, the deviation of *n* from 1 can pass unnoticed, resulting in a significant bias in reported results.

**Fig 5:**
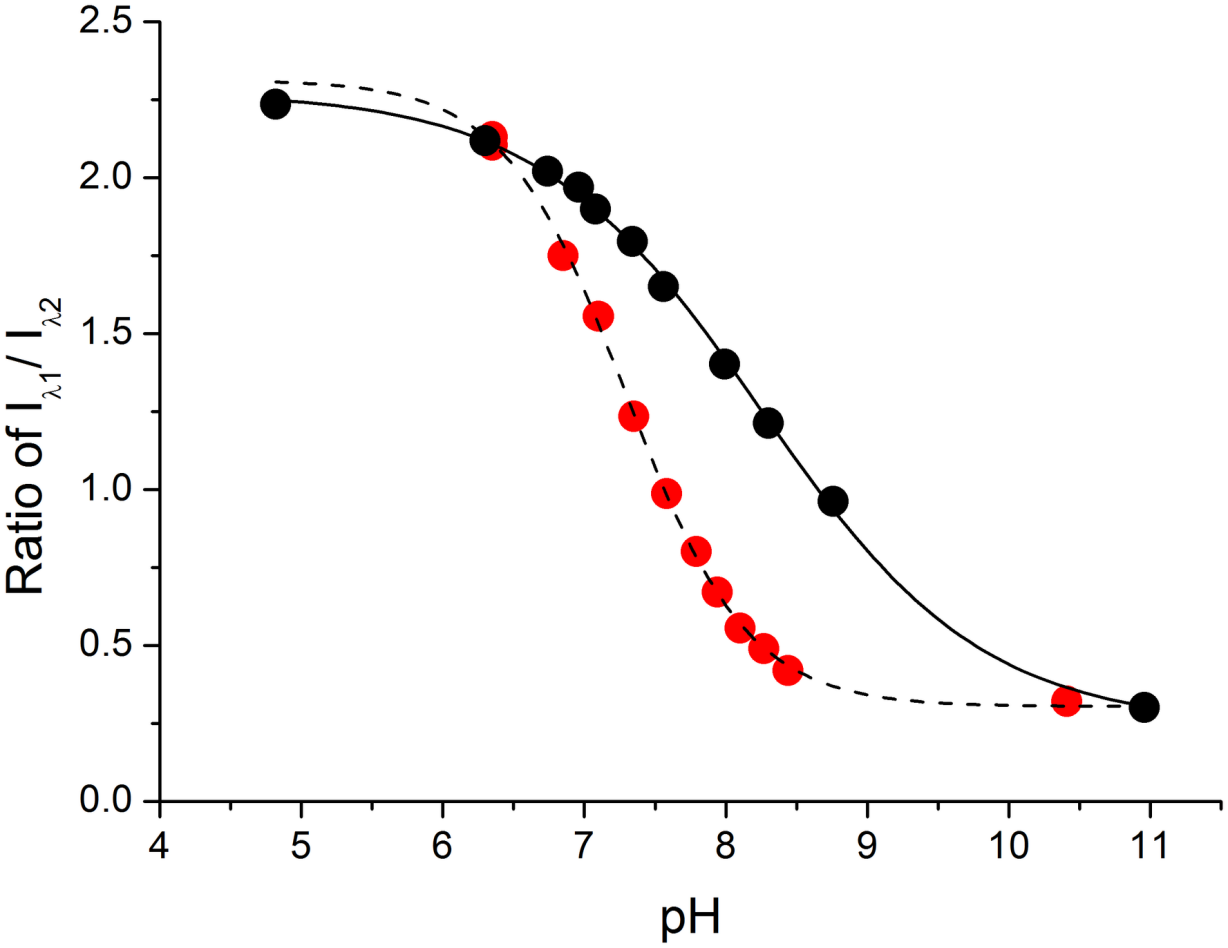
Comparison of the carboxy-SNARF-1 calibration *in vitro* in a pH-buffered solution (red) and in isolated mitochondria (black).

These issues, fundamental for the usage of pH sensors in biological systems, are clearly illustrated in Fig 5, where the calibration curve obtained in buffered solutions exhibits no cooperativity (*n* = 1) and the p*K*_a_ can be calculated using any one of the presented equations, providing that appropriate actions were undertaken to minimize the influence of non-linear ratio effects (Eqs 9 and 10). However, when calibration was performed in mitochondria using an ionophore to equilibrate their internal proton availability with the pH of the solution, a significant negative cooperativity was clearly observed (*n* = 0.54). This fact clearly demonstrates that any calibration of the signal should be performed over a wide range of analyte concentration to see the real probe’s response. The application of three-point linear standard curves, sometimes seen in publications, is likely to lead to false results. Furthermore, the analysis should be performed using appropriate equations, as discussed above. In this context, it should be emphasized that our report is the first ever to consider and demonstrate the importance of the cooperativity coefficient in the pH sensor data analysis.

The Hill coefficient was derived as an empirical tool to describe non-linear concentration effects in dioxygen binding to hemoglobin [23]. Later its usefulness for the analysis of cooperativity in biochemical reactions was established, and more recently it was derived formally for a variety of biophysical and pharmacological models related to interactions among multiple interaction sites [24, 25]. Based on these analytical papers we can state that the *n* value so significantly different from 1 cannot be explained in the framework of a simple proton exchange reaction presented in Eq 1 and, instead, must result from an anticooperative interaction within a bimolecular or multimolecular complex. This fact corresponds very well with our recent conceptual work outlined in the Introduction section, where we discussed the availability of H^+^ ions in biological compartments in general and in mitochondria in particular [1]. According to this view, in the mitochondria, due to their very small size, the availability of free H^+^ ions derived from water autodissociation is negligibly low. Instead, the H^+^ ions feeding the proton pump and supplying any acid/base processes would have to be delivered by reversibly protonated molecules–“proton chaperones”. We suggested that such role could be played by membrane phospholipids and/or soluble phosphates. Of course, the same principle is applicable to any proton exchanging molecules entering the mitochondria, such as carboxy-SNARF-1. The negative cooperativity demonstrated by our experiments can be therefore regarded as indirect, but strong evidence for this concept.

Further evidence in favor of our idea can be in fact found when we confront our data with the previous literature. The average pH in mitochondria determined by us with the sensor cooperativity issue taken into account was 7.1. This value is apparently at odds with the current concept on the gradient of protons across the inner membrane, which is the basis of the paradigm of mitochondrial function. In several previous publications, where carboxy-SNARF-1 was used to measure pH in mitochondria in mammalian cell lines, values between 7.7 and 8 were reported, in accord with the general literature consensus [26]. This probe was not used previously to measure pH in yeast mitochondria, but such data are available from a study using a genetically encoded pH sensor—pHluorin, which yielded a somewhat lower value, 7.5 [27]. When, however, we calculated the mitochondrial pH according to the manufacturer’s instructions, like our predecessors, then the “paradigm” values, 7.7–7.8 were obtained. The difference results solely from the inclusion of the cooperativity factor in the analysis. Therefore, the data published by Orij et al., in fact, support our results [27]. They presented a comparison of the pHluorin calibration in the cytosol and mitochondria. The same pH range was used for both titrations, but the titration end-points are visible in their data for the case of cytosol, and not for the case of mitochondria. This apparent discrepancy strongly suggests the presence of a negative cooperativity in the latter case, exactly as we observed in our experiments. According to our analysis, eukaryotic cytosolic compartment is large enough to maintain acid-base chemistry similar to that of the bulk solution [1]. Furthermore, similar observations can be made by analyzing the published data for the cytosol and mitochondria of the MDCK cell line where carboxy-SNARF-1 was used [8]. In that publication, the pH was calculated from just four experimental points in a narrow pH range, using a linear equation. Applying this approach for example to the first set of our data (with the pH range limited to 7.56–8.56) we can obtain the mitochondrial pH of 7.45, more than 0.3 pH units higher than the value we consider to be correct, but in an excellent accord with the results of Orij et al. [27]. Such discrepancy is not limited to the carboxy-SNARF-1 probe, but can also be observed in the case of fluorescent proteins [28, 29]. On the other hand, the recent pH measurement in the mitochondrial matrix using a locally expressed mitoSypHer sensor protein yielded ‘standard’ pH values of 7.6–7.8 with n values around 0.9 [30]. It should be noted, however, that in those calculation the non-linear effects of ratio calculation were not taken into account, and with a different approach to calibration, recommended in our previous study, the value might be actually close to one determined in this work [20]. It is also possible that genetically encoded and small molecule sensors might be influenced by the measurement medium in a different way. It seems that the issue of pH measurements in the mitochondria and other small volume compartments should be carefully reconsidered.

## Conclusions

To conclude, it appears that the “paradigm” alkaline pH inside the mitochondria is an artifact of the common but erroneous approach to calibration of fluorescent probes, which ignores the negatively cooperative behavior of calibration curves recorded in the organelles. Although this result is in an apparent contradiction with the well-established mechanism of energy generation in the mitochondria, one should take into account that, as we pointed out in our previous work, the very definition of pH in small compartments, such as the mitochondria is hardly relevant. Altogether, our results in conjunction with the previous work [1, 20] suggest that the molecular mechanisms behind the eventual proton gradient in the mitochondria will have to be re-established. Our results also suggest that common views regarding the pH inside other small organelles, for example lysosomes, should be challenged experimentally, using proper probe calibration procedures.

## Appendix

### Formulae for correct fitting of ratiometric data, ready to use in Origin and Matlab

**1. For the ratios of I_1_/I_2_ (R_12_), according to** Eq 17**:**

y=((I1b*Z^n)+(I1u*K^n))/((I2b*Z^n)+(I2u*K^n))

Parameters:K=10^-(pk);                         Z=10^(-x);

where x is pH; y is fluorescence ratio intensity (R_12_); I1b and I2b are the intensities of fluorescence at λ1 and λ2, respectively at basic conditions and I1a and I2a are the intensities of fluorescence at λ1 and λ2, respectively at acidic conditions; *n* is the Hill’s coefficient.

Explicit version of R_12_ (without K and Z parameters)

y=((I1b*(10^(-x*n)+(I1u*(10^-(pK*n))/((I2b*(10^(-x*n)+(I2u*(10^-(pK*n))

**2. For the ratios of I_2_/I_1_ (R_21_), according to** Eq 18**:**

y=((I2b*Z^n)+(I2u*K^n))/((I1b*Z^n)+(I1u*K^n))K=10^(-pk);Z=10^(-x);

Explicit version of R_21_ (without K and Z parameters)

y=((I2b*(10^(-x*n)+(I2u*(10^(-pK*n))/((I1b*(10^(-x*n)+(I1u*(10^(-pK*n))

The equations have the same annotation as above.

## Supporting Information

S1 Fig Three sets of pH calibration of experiments in mitochondria from three separate isolations. The same pH values for calibration buffers were used in all three cases: 4.82, 6.30, 6.74, 6.96, 7.08, 7.34, 7.56. 7.99, 8.30, 8.76, 10.96.
(TIF)
