# Supplementary figures and images for: Revisiting Mitochondrial pH with an Improved Algorithm for Calibration of the Ratiometric 5(6)-carboxy-SNARF-1 Probe Reveals Anticooperative Reaction with H+ Ions and Warrants Further Studies of Organellar pH
Source: PLoS One. 2016 Aug 24;11(8):e0161353. doi: 10.1371/journal.pone.0161353 (PMC4996429; doi:10.1371/journal.pone.0161353)

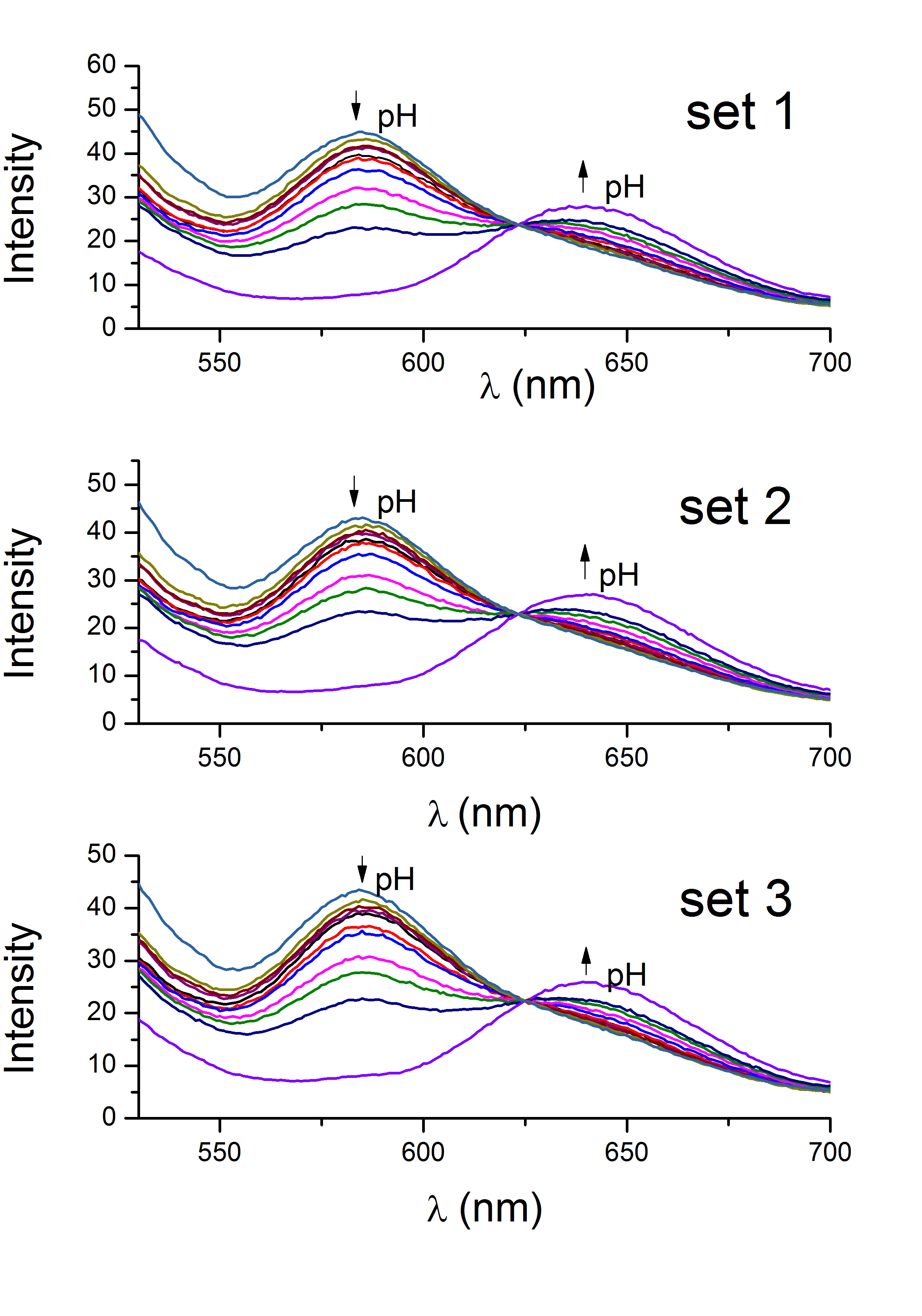

Supplement: S1 Fig — The same pH values for calibration buffers were used in all three cases: 4.82, 6.30, 6.74, 6.96, 7.08, 7.34, 7.56. 7.99, 8.30, 8.76, 10.96. (TIF) [file pone.0161353.s001.tif]
